# The NICE Evidence Standards Framework for digital health and care technologies – Developing and maintaining an innovative evidence framework with global impact

**DOI:** 10.1177/20552076211018617

**Published:** 2021-06-24

**Authors:** Harriet Unsworth, Bernice Dillon, Lucie Collinson, Helen Powell, Mark Salmon, Tosin Oladapo, Lynda Ayiku, Gary Shield, Joanne Holden, Neelam Patel, Mark Campbell, Felix Greaves, Indra Joshi, John Powell, Alexia Tonnel

**Affiliations:** 1 Department of Computer Science, University of Liverpool, UK; 2 Centre for Health Technology Evaluation, NICE, Manchester, UK; 3 Digital, Information and Technology Directorate, NICE, Manchester, UK; 4 Health and Social Care Directorate, NICE, Manchester, UK; 5 MedCity, London, UK; 6 Science, Evidence and Analytics Directorate, NICE, Manchester, UK; 7 NHSx, London, UK; 8 Nuffield Department of Primary Care Health Sciences, University of Oxford, Oxford, UK

**Keywords:** Health economics, health technology assessment, digital health, general, clinical evidence

## Abstract

**Objective:**

In 2018, the UK National Institute for Health and Care Excellence (NICE), in partnership with Public Health England, NHS England, NHS Improvement and others, developed an evidence standards framework (ESF) for digital health and care technologies (DHTs). The ESF was designed to provide a standardised approach to guide developers and commissioners on the levels of evidence needed for the clinical and economic evaluation of DHTs by health and care systems.

**Methods:**

The framework was developed using an agile and iterative methodology that included a literature review of existing initiatives and comparison of these against the requirements set by NHS England; iterative consultation with stakeholders through an expert working group and workshops; and questionnaire-based stakeholder input on a publicly available draft document.

**Results:**

The evidence standards framework has been well-received and to date the ESF has been viewed online over 55,000 times and downloaded over 19,000 times.

**Conclusions:**

In April 2021 we published an update to the ESF. Here, we summarise the process through which the ESF was developed, reflect on its global impact to date, and describe NICE’s ongoing work to maintain and improve the framework in the context for a fast moving, innovative field.

## Introduction

Digital health technologies (DHTs) comprise a wide range of products including apps, software and online platforms that are intended to benefit people or the wider health and care system. The rapid emergence of DHTs presents both opportunities and challenges to health systems seeking new ways to deliver effective, lower cost, patient-centred care at scale.^1^ The pace of development in this field has meant that many DHTs are being introduced to the market with little evidence to show effectiveness, and no standardised evaluation by healthcare systems. Robust health technology assessment (HTA) is used to identify the most effective interventions and ensures the most efficient use of resources. However, traditional HTA relies on the appraisal of published evidence, which is time- and resource-intensive to generate and doesn’t fit the rapid development cycles of DHTs. Requirements for published clinical and economic studies are often described as a barrier to innovations that have the potential to drive service improvements, bring transformational change to health and care delivery, and offer wider economic benefits.^2–5^

In light of this perceived conflict between traditional HTA methods and the rapid nature of DHT development, some have argued that the HTA of DHTs should use a different level and type of evidence to other interventions.^6^ A number of initiatives have attempted to create frameworks for evaluating DHTs.^7^ A review of evaluation frameworks for mobile medical applications by Moshi et al. identified 45 frameworks published between 2011 and 2016 which sought to guide health technology assessment of these tools.^8^

In this context, NHS England commissioned the UK National Institute for Health and Care Excellence (NICE) to develop a proportional evidence standards framework (ESF) for DHTs, to include both effectiveness and economic impact considerations.^9^ The aim of the ESF is to provide NHS England health and care commissioners with a tool to understand what ‘good evidence’ looks like – what kind of evidence is needed to show that DHTs are likely to be clinically- and cost-effective. In addition, it can help developers to understand what kind of evidence they should develop for their DHT to be used in the UK health and care system.

The ESF was created to underpin principle 8 of the Code of Conduct for Data-Driven Health and Care Technology published by the UK Department of Health and Social Care. The code of conduct outlines 10 principles to guide the development and implementation of DHTs in the UK health and care system.^10^ It complements other initiatives including NHSX’s Digital Technology Assessment Criteria (DTAC),^11^ which was launched in Spring 2021 to replace NHS Digital’s digital assessment questionnaire (DAQ).^12^ The DTAC covers 5 core areas: clinical safety, data protection, technical assurance, interoperability, and usability and accessibility.

In this paper, we describe the agile policy research approach used to develop the ESF, outline how the ESF works, and describe its impact to date and ongoing work to ensure that the ESF remains up to date with the rapidly changing field of digital healthcare.

## Methods

A core working group was established to develop the NICE ESF. This included representatives from NICE, NHS England, Public Health England and the health and life science cluster organisation MedCity. Additional stakeholders included NHS Digital, the UK Office for Life Sciences, National Institute for Health Research, British Standards Institute, Innovate UK and Digital Health London.

NICE were given the following criteria for developing the ESF:

- The ESF was not intended to be used for DHTs that incorporate artificial intelligence using adaptive algorithms (those which continuously evolve), although it would cover tools that use fixed algorithms (those which do not change within the commissioning period, or those which have periodic updates to release iterations of the algorithm).
- The ESF was not intended to cover safety issues nor to be a regulatory tool: in the UK, the regulation of DHTs that are categorised as medical devices is governed by the Medicines and Healthcare products Regulatory Agency (MHRA).
- NHS England set the following requirements that the ESF should:
  1. be suitable for use by health and care commissioners and people who are not expert in HTA, clinical matters or digital information technology
  2. be sufficiently comprehensive to cover the range of DHTs that are most often commissioned in the UK health and care system
  3. take account of the current evidence levels available for digital tools across the spectrum of DHT functions
  4. include defined standards of evidence that must be met for commissioning in the UK health and care system
  5. fit alongside other existing regulation in the UK without duplication or omission of factors
  6. include some means to assess the economic and system-level impacts of DHTs.

The required timeline for the development of the framework necessitated an agile and iterative methodology. This involved: a literature review of existing initiatives and comparison of these against the requirements set by NHS England, iterative consultation with stakeholders through an expert working group and series of workshops, and questionnaire-based stakeholder input on a draft document which was made publicly available.

### Literature searches

Literature searches were carried out to identify published methods for classifying and evaluating DHTs. Database searches were conducted via the Ovid platform in MEDLINE, MEDLINE In-Process, MEDLINE ePub ahead of print, MEDLINE daily update, Embase and the Health Management Information Consortium (HMIC) database in May 2018, when the first version of the ESF was developed. MeSH terms in combination with free text searches for keywords in the title were used. In addition, website searches were conducted to identify grey literature in May 2018.

Potentially relevant papers were reviewed to assess whether they presented a framework for the evaluation of DHTs, and in particular whether any frameworks met the 6 requirements which had been specified by NHS England.

### Expert workshops to develop and iterate the draft ESF

The ESF was drafted by the core working group based on the literature searches and using NICE’s experience of evaluating medical devices and DHTs. The draft ESF was then repeatedly iterated based on feedback received from a series of workshops.

In total, 13 workshops were held between June and December 2018 with representatives of the medtech and DHT industry, health and care commissioners, academic HTA experts, clinicians and others. Over 150 people from 95 organisations, including universities, industry, National Institute for Health Research, NHS Trusts, National Associations, Royal Colleges, Department of Health and Social Care, NHS Digital, NHS Improvement, private health insurers, Office for Life Sciences and Public Health England, took part. In addition to the workshops, the draft ESF was shared directly with key stakeholder organisations, such as the MHRA, for comment.

11 of the 13 workshops were held to develop the content of the ESF, and the final 2 were held to assess the usability of the final ESF. The usability workshops were attended by innovators and developers from 31 companies, 11 of which had been represented in the ESF development workshops.

### Publication of the beta version of the ESF

In December 2018, a beta version of the ESF was published on the NICE website. Scrutiny, comment and feedback on the beta version were invited from stakeholders and the public, via direct email to stakeholders who had expressed an interest during the workshops or were identified by the project team as having relevant experience and expertise. In addition, an extensive marketing, social media and communication campaign was coordinated by MedCity, who produced a video describing the ESF project. This approach was seen to be particularly relevant for small to innovators in medium enterprises (SMEs) and international stakeholders. NICE staff and other core working group members presented the ESF at over 20 relevant industry and healthcare events to gather verbal feedback and an online survey was used for written feedback. The survey included dichotomous, Likert scale and free text questions and was open for one month after the publication of the draft framework. Numerical responses were analysed using frequencies and free text responses were grouped thematically to identify common concerns.

After consideration of the feedback on the beta version, ESF version 1.0 was published in March 2019. Alongside the ESF document we published the following supporting materials that had been developed by the NICE team and academic collaborators:

- A user guide to explain the concepts and terms used in the ESF
- A budget impact template to help users to develop a budget impact model
- A case studies document describing real DHTs that were already in use in the NHS, their functional classification and how they were already meeting the evidence standards
- A functional classification document describing how over 90 real DHTs would be classified according to the ESF functional classification.

### Post-launch stakeholder feedback

A second round of stakeholder feedback was sought in September 2019 to ascertain whether the ESF was meeting the needs of users, and to collect suggestions for improving the ESF and its supporting documents.

An online survey was created and linked from the ESF pages of the NICE website. Key stakeholders were contacted by email to encourage participation in the survey. The survey included dichotomous, Likert scale and free text questions and was open for 6 weeks. Analysis of the survey responses was performed as before. In addition, interviews were held with key stakeholders to obtain their views on the ESF.

## Results

### Literature searches

The search for existing frameworks yielded a total of 4877 hits when it was conducted in March 2018. An initial sift for relevant titles identified 299 potentially relevant articles, and from these we identified 8 published frameworks which at least partially met the requirements set by the policy commissioners for the evaluation of effectiveness (see Table 1).^13–20^ None of the 8 shortlisted systems met all 6 requirements.

**Table 1.** Summary of the eight published frameworks identified and comparison with the requirements set by the policy commissioners for the evaluation of effectiveness.

Requirement	Published protocol for evaluating DHTs							
	Nielsen and Rimpilainen^14^	Sadegh et al.^15^	Betton et al.^16^	Baumel et al.^17^	Murray et al.^13^	Grundy et al.^18^	Stoyanov et al.^19^	Lewis and Wyatt^20^
Suitable for use by commissioners: • Can be used by non-HTA experts • Can be used by non-clinical experts • Can be used by non-IT experts	No	Yes	No	No	Yes	Yes	Yes	Varies depending on risk level
Covers the range of DHTs expected to be most frequently purchased or commissioned in UK health and care system	Yes	Yes	No	No	No	No	No	Yes
Is reflective of the current evidence levels available for DHTs across the spectrum of function	Yes	Yes	Yes	Yes	No	Yes	Yes	Yes
Includes defined standards of evidence that must be met	No	No	No	No	No	No	No	No
Fits alongside other existing regulation for DHTs in the UK health and social care system without duplication or omission of factors	Yes	No	Yes	No	Yes	Yes	Yes	No
Includes some means to assess system and economic impact of DHTs	No	Yes	Yes	No	Yes	No	No	No

The review of the literature also identified 3 approaches to classifying DHTs based on their functionality.

Firstly, the World Health Organization (WHO) Classification of Digital Health Interventions uses 87 functional categories split into 4 end user groups.^21^ This classification is easy to understand but has multiple categories that were outside our remit. It also lacked granularity for DHTs which provide treatment or diagnosis. Secondly, the FDA used the International Medical Device Regulators Forum (IMDRF) principles to approach the clinical evaluation of DHTs.^22^ This model stratifies the level of independent review needed, according to the level of risk. Risk is based on the function of the software (inform clinical management, drive clinical management, or treat/diagnose) and the severity of the healthcare situation or condition (non-serious, serious, critical). We concluded that more than 3 functional groups would be needed to cover the breadth of DHTs included in our remit, and that the function of the DHT may be a more suitable driver for risk rather than the health condition. Finally, at the time of writing the UK MHRA classified digital and non-digital medical devices and diagnostics according to risk in line with the EU Medical Devices Directive CE marking system, in which higher-risk devices (classes IIa, IIb and III) had higher evidence requirements.^24^ However, CE marking is designed to regulate the safety of medical devices, whereas our framework is intended to demonstrate effectiveness. Also, the remit for the ESF includes many DHTs that fall outside the current CE marking classifications. Therefore, it was concluded that aligning the ESF to the CE marking classifications would not be useful in practice.

### Developing the ESF: evidence of effectiveness standards

**Figure 1.**
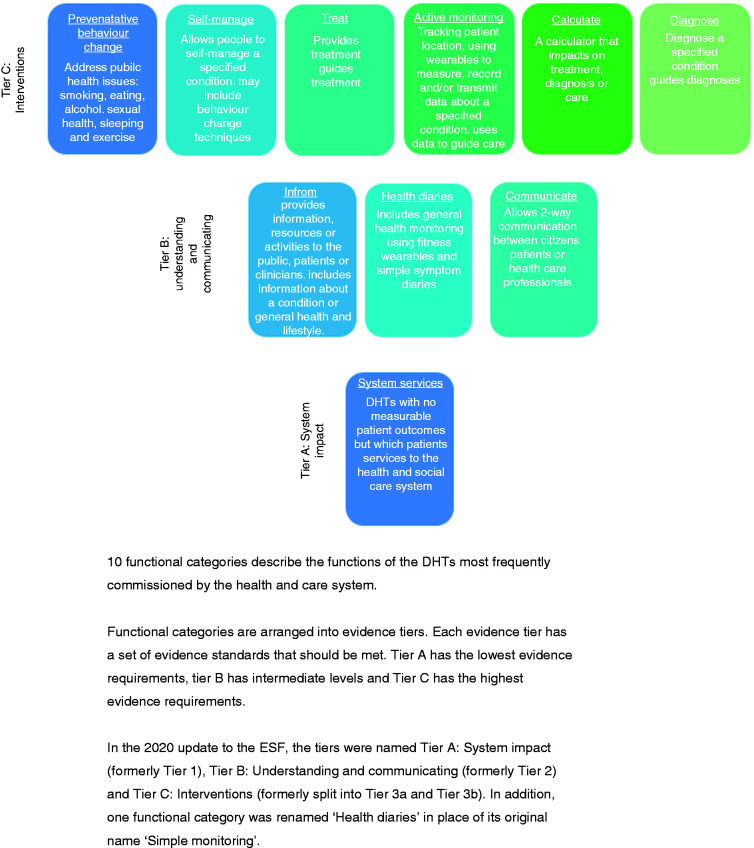
DHTs classified by function and stratified into evidence tiers.

Based on the literature searches and feedback from stakeholder workshops, we developed a bespoke taxonomy with 10 functional categories that are expected to cover the functions of the majority of the DHTs most frequently commissioned in the UK health and care system (Table 2 and Figure 1). This approach was grounded in the concept that the quality and quantity of evidence should be proportionate to the potential clinical risk and the financial and impact of the DHT.

**Table 2.** Functional classification of DHTs.

Evidence tier	Functional classification	Description	Includes (for example)	Excludes (for example)
Tier A: System impact DHTs with potential system benefits but no direct user benefits.	System service	Improves system efficiency. Unlikely to have direct and measurable individual patient outcomes.	Electronic prescribing systems that do not provide patient-level advice on prescribing. Electronic health record platforms. Ward management systems.	CE marked medical devices and systems that provide treatment or diagnoses, such as early warning systems that monitor patient vital signs.
Tier B: Understanding and communicating DHTs which help users to understand healthy living and illnesses but are unlikely to have measurable user outcomes.	Inform	Provides information and resources to patients or the public. Can include information on specific conditions or about healthy living.	DHTs describing a condition and its treatment. Apps providing advice for healthy lifestyles (such as recipes). Apps that signpost to other services.	Tools that collect symptom data from users. Tools that provide treatment for a condition. DHTs that allow communication among users, or between users and professionals.
	Health diaries	Allows users to record health parameters to create health diaries. This information is not shared with or sent to others.	Health tracking information such as from fitness wearables. Symptom or mood diaries.	DHTs that share information with professionals, carers or other users. Tools that provide treatment for a condition.
	Communicate	Allows 2-way communication between users and professionals, carers, third-party organisations or peers. Clinical advice is provided by a professional using the DHT, not by the DHT itself.	Instant messaging apps for health and social care. Video conference-style consultation software. Platforms for communication with carers or professionals.	DHTs that provide clinical content themselves (such as cognitive behavioural programmes for depression).
Tier C: Interventions DHTs for preventing, diagnosing and managing diseases. They may be used alongside other treatments and will likely have measurable user benefits. DHTs in the Treat, Active monitoring, Calculate or Diagnose categories will likely be CE-marked medical devices	Preventative behaviour change	Designed to improve health behaviours to prevent ill-health consequences associated with smoking, eating, alcohol use, sexual health, sleeping and exercise. Based on accepted behaviour change theories.	Smoking cessation DHTs and those used as part of weight loss programmes. DHTs marketed as aids to good sleep habits.	DHTs that describe themselves as a treatment for a diagnosed condition. Apps that provide general healthy lifestyle advice.
	Self-manage	Aims to help people with a diagnosed condition to manage their health. May include symptom tracking function that connects with a healthcare professional. May be based on accepted behaviour change theories.	DHTs that allow users to record, and optionally to send, data to a healthcare professional to improve management of their condition.	DHTs that describe themselves as a treatment for a diagnosed condition. Apps that automatically monitor and report data to a healthcare professional or third-party organisation.
	Treat	Provides treatment for a diagnosed condition (such as CBT for anxiety), or guides treatment decisions.	DHTs for treating mental health or other conditions. Clinician-facing apps that advise on treatments in certain situations. Electronic prescribing systems that provide patient-level advice on prescribing.	DHTs that provides general health advice or advice on living with a diagnosed condition. DHTs that offer general advice for clinicians such as online textbooks or digital versions of care pathways.
	Active monitoring	Automatically records information and transmits the data to a professional, carer or third-party organisation, without any input from the user, to inform clinical management decisions. Uses data to guide care or treatment.	DHTs linked to devices such as implants, sensors worn on the body, or sited in the home or care setting, where data are automatically transmitted for remote monitoring. Includes ward-based systems for monitoring and recording patient observations.	DHTs that allow a user to choose if and when to send recorded data to a professional, carer or third-party organisation.
	Calculate.	Tools that perform clinical calculations that are likely to affect clinical care decisions.	DHTs for use by clinicians, professionals or users to calculate parameters pertaining to care, such as early warning system software.	DHTs that diagnose or provide treatment for a condition.
	Diagnose.	Uses data to diagnose a condition in a patient, or to guide a diagnostic decision made by a healthcare professional.	DHTs that diagnose specified clinical conditions using clinical data.	DHTs that offer general lists of signs and symptoms for healthcare conditions.

The 10 functional categories are arranged into ‘tiers’ according to the level of potential risk associated with that function. We use the term ‘potential risk’ to describe level of harm to the user which could arise for example from unintended negative consequences for the user’s health and wellbeing from using the DHT. Some DHTs may fit into several functional categories and in this case the highest risk category should be used.

Each tier within the functional classification is linked to a set of evidence standards that are designed to be appropriate to the risk and impact for that level of the classification (Tables 3 [Table table4-20552076211018617]to 5). The level of evidence needed for each tier is proportionate to the potential risk to users presented by the DHT, whereby the higher levels of evidence are needed for the higher risk tiers. For example, published evidence is not required for the basic tier, but for the highest tier published comparative studies are needed.

**Table 3.** Evidence for effectiveness standards for Tier A: system impact DHTs.

Evidence category	Minimum evidence standard	Best practice standard
Credibility with UK health and social care professionals.	Be able to show that the DHT has a plausible mode of action that is viewed as useful and relevant by professional experts or expert groups in the relevant field. Either: • show that relevant clinical or social care professionals working within the UK health and social care system have been involved in the design, development or testing of the DHT, or • show that relevant clinical or social care professionals working within the UK health and social care system have been involved in signing-off the DHT, indicating their informed approval of the DHT.	Published or publicly available evidence documenting that the DHT has a plausible mode of action that is viewed as useful and relevant by professional experts or expert groups in the relevant field. Either: • show that relevant clinical or social care professionals working within the UK health and social care system have been involved in the design, development or testing of the DHT, or • show that relevant clinical or social care professionals working within the UK health and social care system have been involved in signing-off the DHT, indicating their informed approval of the DHT..
Relevance to current care pathways in the UK health and social care system.	Evidence to show that the DHT has been successfully piloted in the UK health and social care system, showing that it is relevant to current care pathways and service provision in the UK. Also, evidence that the DHT can perform its intended function to the scale needed (for example, having servers that can scale to manage the expected number of users).	Evidence to show successful implementation of the DHT in the UK health and social care system.
Acceptability with users.	Be able to show that representatives from intended user groups were involved in the design, development or testing of the DHT. Provide data to show user satisfaction with the DHT.	Published or publicly available evidence to show that representatives from intended user groups were involved in the design, development or testing of the DHT and to show that users are satisfied with the DHT.
Equalities considerations.	Evidence, if relevant, that the DHT: • Contributes to challenging health inequalities in the UK health and social care system, or improving access to care among hard-to-reach populations. • Contribute to promoting equality, eliminating unlawful discrimination and fostering good relations between people with protected characteristics (as described in the 2010 Equalities Act) and others.	Show evidence of the DHT being used in hard-to-reach populations, or that its use reduces health inequalities.
Accurate and reliable measurements (if relevant).	Data or analysis which shows that the data generated or recorded by the DHT is: • accurate • reproducible • relevant to the range of values expected in the target population. Also data showing that the DHT is able to detect clinically relevant changes or responses.	As for the minimum evidence standard, but with quantitative data.
Accurate and reliable transmission of data (if relevant).	Technical data showing that numerical, text, audio, image-based, graphic-based or video information is: • not changed during the transmission process • not biased by the data ‘value’ expected from the target patient population.	As for the minimum evidence standard, but with quantitative data.

**Table 4. table4-20552076211018617:** Evidence for effectiveness standards for Tier B: understanding and communicating DHTs.

Evidence category	Minimum evidence standard	Best practice standard
Reliable information content.	Be able to show that any health information provided by the DHT is: • valid (aligned to best available sources, such as NICE guidance, relevant professional organisations or recognised UK patient organisations, and appropriate for the target population) • accurate • up to date • reviewed and updated by relevant experts at defined intervals, such as every year • sufficiently comprehensive.	Evidence of endorsement, accreditation or recommendation by NICE, NHS England, a relevant professional body or recognised UK patient organisation. Alternatively, evidence that the information content has been validated though an independent accreditation.
Ongoing data collection to show usage of the DHT.	Commitment to ongoing data collection to show *usage* of the DHT in the target population, and commitment to share, when available, with relevant decision-makers such as commissioners in a clear and useful format.	Evidence that data on *usage* is being collected in line with the minimum standards and can be made available to relevant decision-makers.
Ongoing data collection to show value of the DHT.	Commitment to ongoing data collection to show *user outcomes* (if relevant) or *user satisfaction* (using non-patient identifiable information) to show ongoing value, and commitment to share, when available, with relevant decision-makers such as commissioners in a clear and useful format.	Evidence that data on *outcomes or user satisfaction* is being collected in line with the minimum standard and can be made available to relevant decision-makers.
Quality and safeguarding.	Show that appropriate safeguarding measures are in place around peer-support and other communication functions within the platform. Describe: • who has access to the platform and their roles within the platform • why these people or groups are suitable and qualified to have access • any measures in place to ensure safety in peer-to-peer communication, for example through user agreements or moderation.	As for the minimum evidence standard.
Credibility with UK health and social care professionals.	Be able to show that the DHT has a plausible mode of action that is viewed as useful and relevant by professional experts or expert groups in the relevant field. Either: • show that relevant clinical or social care professionals working within the UK health and social care system have been involved in the design, development or testing of the DHT, or • show that relevant clinical or social care professionals working within the UK health and social care system have been involved in signing-off the DHT, indicating their informed approval of the DHT.	Published or publicly available evidence documenting that the DHT has a plausible mode of action that is viewed as useful and relevant by professional experts or expert groups in the relevant field. Either: • show that relevant clinical or social care professionals working within the UK health and social care system have been involved in the design, development or testing of the DHT, or • show that relevant clinical or social care professionals working within the UK health and social care system have been involved in signing-off the DHT, indicating their informed approval of the DHT.
Relevance to current care pathways in the UK health and social care system.	Evidence to show that the DHT has been successfully piloted in the UK health and social care system, showing that it is relevant to current care pathways and service provision in the UK. Also, evidence that the DHT can perform its intended function to the scale needed (e.g., having servers that can scale to manage the expected number of users).	Evidence to show successful implementation of the DHT in the UK health and social care system.
Acceptability with users.	Be able to show that representatives from intended user groups were involved in the design, development or testing of the DHT. Provide data to show user satisfaction with the DHT.	Published or publicly available evidence to show that representatives from intended user groups were involved in the design, development or testing of the DHT and to show that users are satisfied with the DHT.
Equalities considerations.	Evidence, if relevant, that the DHT: • Contributes to challenging health inequalities in the UK health and social care system, or improving access to care among hard-to-reach populations. • Contribute to promoting equality, eliminating unlawful discrimination and fostering good relations between people with protected characteristics (as described in the 2010 Equalities Act) and others.	Show evidence of the DHT being used in hard-to-reach populations, or that its use reduces health inequalities.
Accurate and reliable measurements (if relevant).	Data or analysis which shows that the data generated or recorded by the DHT is: • accurate • reproducible • relevant to the range of values expected in the target population. Also, data showing that the DHT is able to detect clinically relevant changes or responses.	As for the minimum evidence standard, but with quantitative data.
Accurate and reliable transmission of data (if relevant).	Technical data showing that numerical, text, audio, image-based, graphic-based or video information is: • not changed during the transmission process • not biased by the data ‘value’ expected from the target patient population.	As for the minimum evidence standard, but with quantitative data.

**Table 5.** Evidence for effectiveness standards for Tier C: Intervention DHTs.

Evidence category	Minimum evidence standard	Best practice standard
Demonstrating effectiveness – for preventative behaviour change or self-manage functions	High quality observational or quasi-experimental studies demonstrating relevant outcomes. These studies should present comparative data. Comparisons could include: • relevant outcomes in a control group • use of historical controls • routinely collected data. Relevant outcomes may include: • behavioural or condition-related user outcomes such as reduction in smoking or improvement in condition management • evidence of positive behaviour change • user satisfaction.	High quality intervention study (quasi-experimental or experimental design) which incorporates a comparison group, showing improvements in relevant outcomes, such as: • patient-reported outcomes (preferably using validated tools) including symptom severity or quality of life • other clinical measures of disease severity or disability • healthy behaviours • physiological measures • user satisfaction and engagement • health and social care resource use, such as admissions or appointments. The comparator should be a care option that is reflective of standard care in the current care pathway, such as a commonly used active intervention.
Demonstrating effectiveness *for Treat, Active monitoring, Calculate or Diagnose functions*	High quality intervention study (experimental or quasi-experimental design) showing improvements in relevant outcomes, such as: • diagnostic accuracy • patient-reported outcomes (preferably using validated tools) including symptom severity or quality of life • other clinical measures of disease severity or disability • healthy behaviours • physiological measures • user satisfaction and engagement. Generic outcome measures may also be useful when reported alongside condition-specific outcomes. The comparator should be a care option that is reflective of the current care pathway, such as a commonly used active intervention.	High quality randomised controlled study or studies done in a setting relevant to the UK health and social care system, comparing the DHT with a relevant comparator and demonstrating consistent benefit including in clinical outcomes in the target population, using validated condition-specific outcome measures. Alternatively, a well-conducted meta-analysis of randomised controlled studies if there are enough available studies on the DHT.
Use of appropriate behaviour change techniques (if relevant).	Be able to show that the techniques used in the DHT are: • consistent with recognised behaviour change theory and recommended practice (aligned to guidance from NICE or relevant professional organisations) • appropriate for the target population.	Published qualitative or quantitative evidence showing that the techniques used in the DHT are: • based on published and recognised effective behaviour change techniques • aligned with recommended practice • appropriate for the target population.
Reliable information content.	Be able to show that any health information provided by the DHT is: • valid (aligned to best available sources, such as NICE guidance, relevant professional organisations or recognised UK patient organisations, and appropriate for the target population) • accurate • up to date • reviewed and updated by relevant experts at defined intervals, such as every year • sufficiently comprehensive.	Evidence of endorsement, accreditation or recommendation by NICE, NHS England, a relevant professional body or recognised UK patient organisation. Alternatively, evidence that the information content has been validated though an independent accreditation such as The Information Standard or HONcode certification.
Ongoing data collection to show usage of the DHT.	Commitment to ongoing data collection to show *usage* of the DHT in the target population, and commitment to share, when available, with relevant decision-makers such as commissioners in a clear and useful format.	Evidence that data on *usage* is being collected in line with the minimum standards and can be made available to relevant decision-makers.
Ongoing data collection to show value of the DHT.	Commitment to ongoing data collection to show *user outcomes* (if relevant) or *user satisfaction* (using non-patient identifiable information) to show ongoing value, and commitment to share, when available, with relevant decision-makers such as commissioners in a clear and useful format.	Evidence that data on *outcomes or user satisfaction* is being collected in line with the minimum standard and can be made available to relevant decision-makers.
Quality and safeguarding.	Show that appropriate safeguarding measures are in place around peer-support and other communication functions within the platform. Describe who has access to the platform and their roles within the platform. Describe why these people or groups are suitable and qualified to have access. Describe any measures in place to ensure safety in peer-to-peer communication, for example through user agreements or moderation.	As for the minimum evidence standard.
Credibility with UK health and social care professionals.	Be able to show that the DHT has a plausible mode of action that is viewed as useful and relevant by professional experts or expert groups in the relevant field. Either: • show that relevant clinical or social care professionals working within the UK health and social care system have been involved in the design, development or testing of the DHT, or • show that relevant clinical or social care professionals working within the UK health and social care system have been involved in signing-off the DHT, indicating their informed approval of the DHT.	Published or publicly available evidence documenting that the DHT has a plausible mode of action that is viewed as useful and relevant by professional experts or expert groups in the relevant field. Either: • show that relevant clinical or social care professionals working within the UK health and social care system have been involved in the design, development or testing of the DHT, or • show that relevant clinical or social care professionals working within the UK health and social care system have been involved in signing-off the DHT, indicating their informed approval of the DHT.
Relevance to current care pathways in the UK health and social care system.	Evidence to show that the DHT has been successfully piloted in the UK health and social care system, showing that it is relevant to current care pathways and service provision in the UK. Also evidence that the DHT is able to perform its intended function to the scale needed (for example, having servers that can scale to manage the expected number of users).	Evidence to show successful implementation of the DHT in the UK health and social care system.
Acceptability with users.	Be able to show that representatives from intended user groups were involved in the design, development or testing of the DHT. Provide data to show user satisfaction with the DHT.	Published or publically available evidence to show that representatives from intended user groups were involved in the design, development or testing of the DHT and to show that users are satisfied with the DHT.
Equalities considerations.	Evidence, if relevant, that the DHT: • Contributes to challenging health inequalities in the UK health and social care system, or improving access to care among hard-to-reach populations. • Contribute to promoting equality, eliminating unlawful discrimination and fostering good relations between people with protected characteristics (as described in the 2010 Equalities Act) and others.	Show evidence of the DHT being used in hard-to-reach populations, or that its use reduces health inequalities.
Accurate and reliable measurements (if relevant).	Data or analysis which shows that the data generated or recorded by the DHT is: • accurate • reproducible • relevant to the range of values expected in the target population. Also data showing that the DHT is able to detect clinically relevant changes or responses.	As for the minimum evidence standard, but with quantitative data.
Accurate and reliable transmission of data (if relevant).	Technical data showing that numerical, text, audio, image-based, graphic-based or video information is: • not changed during the transmission process • not biased by the data ‘value’ expected from the target patient population.	As for the minimum evidence standard, but with quantitative data.

The evidence requirements were set according to key evidence areas identified by the NICE team, based on NICE’s experience of evaluating medical devices and DHTs. We took a pragmatic approach of identifying key issues that should be evidenced in order to inform any commissioning decision.

At the most basic level, DHTs need to be based on plausible principles and to be useful in UK clinical and care pathways. People from the intended user groups should have been consulted or included in the development of the DHT, and any equalities issues should have been considered by the developer. Any health information included in the DHT must be accurate and up to date, and safeguarding measures must be in place for any DHTs that allow communication between users. We have asked that developers should collect data to show that the DHT is being used in line with the developer’s and commissioner’s expectations, and to show that it is offering good value to the health and care system. For higher-risk DHTs, we require evidence of effectiveness.

During further testing and feedback, further refinements were made. We developed both minimum (essential) and best practice (desirable) evidence standards for each tier, to allow flexibility for commissioners to stipulate higher evidence levels where specific risks were recognised. We developed a set of contextual questions (Table 6) to identify specific risks, such as DHTs that are designed to be used with people in vulnerable groups. If the contextual questions identify any specific risks with a DHT, the best practice evidence should be met.

**Table 6.** Contextual questions designed to help identify DHTs associated with greater risk to the user.

Question	Risk adjustment
Are the intended users of the DHT considered to be in a potentially vulnerable group such as children or at-risk adults?	NHS England defines an at-risk adult as an adult ‘who may be in need of community care services by reason of mental or other disability, age or illness; and who is or may be unable to take care of him or herself, or unable to protect him or herself against significant harm or exploitation.’ If the DHT is intended to be used by people considered to be in a potentially vulnerable group then a higher level of evidence may be needed, or relevant expert opinion on whether the needs of the users are being appropriately addressed.
How serious could the consequences be to the user if the DHT failed to perform as described?	A higher level of potential harm may indicate that the best practice evidence standards should be used.
Is the DHT intended to be used with regular support from a suitably qualified and experienced health or social care professional?	DHTs that are intended to be used with support (that is, with regular support or guidance from a suitably qualified and experienced health or social care professional) could be considered to have lower risk than DHTs that are intended to be used by the patient on their own. *This contextual question may require careful interpretation depending on the individual DHT as the involvement of a clinician may in itself indicate that the DHT presents a specific risk.*
Does the DHT include machine learning algorithms or artificial intelligence?	Refer to the code of conduct for data-driven health and care technology for additional considerations when assessing DHTs that use artificial intelligence or machine learning.
Is the financial or organisational risk of the DHT expected to be very high?	DHTs with very high financial risk should be assessed using the best practice standards to provide surety that the DHT represents good value. High organisational risks may include situations in which implementing the DHT would need complex changes in working practice or care pathways.

The evidence standards are not designed to replace the requirements for regulatory approval, nor to measure compliance with relevant technical standards for information governance, security, resilience or interoperability.

### Developing the ESF: Evidence of economic impact standards

Our original intention was to integrate the effectiveness and economic impact standards within a single framework, but the resulting matrix was too complex and unclear to users. Instead we developed a separate brief framework for economic impact (Table 7). This took a relatively narrow ‘commissioner’ perspective (i.e. the payer within the health and care system) and we made the pragmatic decision that the level of health economic evidence required would depend on the predicted financial risk to the system of adopting and implementing the DHT, and the potential system benefit of this. The economic evidence standards describe 3 levels of economic evaluation, proportionate to the financial commitment of the health and care system to the DHT.

**Table 7.** Evidence for economic impact standards: appropriate economic analysis.

Economic analysis level	Appropriate economic analysis	Outputs
Basic.	Budget impact analysis.	Estimated yearly budget impact for years 1 to 2. Data may be collected to inform future economic analyses.
Low financial commitment.	Cost–consequence analysis.	Estimated costs and benefits. Sensitivity analysis results.
	Budget impact analysis.	Estimated yearly budget impact for years 1 to 5. Sensitivity analysis results.
High financial commitment.	For DHTs with health outcomes funded by the NHS and Personal Social Services, a cost–utility analysis should be done using NICE's guide to the methods of technology appraisal as a reference case.	Estimated incremental cost–effectiveness ratio. Sensitivity analysis results.
	For DHTs funded by the public sector with health and non-health outcomes, or for DHTs that focus on social care, a cost–utility analysis should be done. If this is not possible, a cost–consequence analysis may be acceptable. The analysis should be done using developing NICE guidelines: the manual as a reference case.	Estimated incremental cost–effectiveness ratio (cost–utility analysis) or estimated costs and benefits (cost–consequence analysis). Sensitivity analysis results.
	Budget impact analysis.	Estimated yearly budget impact for years 1 to 5. Sensitivity analysis results.

The evidence for economic impact standards provide information on the key considerations required for undertaking and reporting economic evaluations of DHTs, as well as categorising the level of financial ‘risk’ into 3 levels: basic, low and high. Typical examples of these 3 levels are, for basic: a pilot project at a local level; for low: a regional initiative or a potentially cost-saving national initiative; and for high: a national commissioning decision likely to be cost-incurring, for example, through service redesign. The type of economic analysis specified in the standards is related to the level of financial risk identified (see Table 7).

### Stakeholder feedback on the beta version of the ESF

The ESF was initially published in beta version on the NICE website on the 10 December 2018. Alongside the ESF and supporting documents was an invitation to stakeholders to provide feedback, with a link to an online survey.

46 complete responses to the feedback survey were received, along with 7 separate written responses. Responses were received from wide range of stakeholders including companies and industry associations, NHS clinicians and managers, digital health academics and national health and care system organisations. At least 80% of respondents who submitted a complete response said that they were clear what the standards were for (93%), who they were aimed at (83%) and how to use them (80%).

The framework was generally welcomed, and most respondents agreed with the content and methods used. The free text comments showed the following concerns:

- the need for greater clarification of terms and definitions; more explanation of how the standards fit within the current regulatory compliance regime and their place in existing market access arrangements
- insufficient recognition of the widespread use of DHTs within social care; a lack of patient and public engagement; difficulties using the framework arising from the dynamic and rapidly evolving nature of digital tools and the likely crossover of technologies between evidence tiers
- a perceived lack of capacity and capability in the system for economic analysis; greater attention needed on the impact of real-world data and real-world evidence
- refinement of the risk concepts and the approach to artificial intelligence. Following this feedback, minor changes were made to the framework to provide additional clarity and specificity.

### Post-launch stakeholder feedback on the ESF

The post-launch feedback survey in September 2019 had 52 responses from stakeholders across industry, academia, patients and clinical experts. Most of the survey respondents had used the framework and overall user experience was positive. Additional feedback was obtained from interviews with a range of stakeholders including digital health specialists, service design consultants and collaborators from partner organisations.

The main themes of the feedback were:

- requesting additional resources to help users understand evidence generation and health economic analyses, a perceived lack of clarity about the remit of the ESF and how it fitted with other initiatives including the NHS Digital DAQ and 2017 EU Medical Device Regulation (MDR) for software
- the need to engage further with commissioners and social care, the need for guidance on the use of real-world evidence in the ESF
- a need for greater clarity in the functional classification system and evidence requirements, requesting information on the role of users in testing and signing off new DHTs
- questions about the process of how the ESF will be used in practice.

Following this feedback, updates to the ESF and supporting documents will be published in April 2021. No major changes were made to the functional classification system, evidence of effectiveness tiers or evidence of economic impact. Some minor updates were made to the evidence of effectiveness standards at this stage:

1. The 3 evidence tiers were renamed in order to make them simpler to understand and to avoid any confusion with CE marking classes. The new tiers, from lowest evidence requirement to highest, are:
  - Tier A: System impact
  - Tier B: Understanding and communicating
  - Tier C: Interventions. This upper tier had previously been split into 2 parts but is now merged into a single tier.
2. The evidence requirements for each evidence tier remain the same but we have changed how they are presented: instead of describing the evidence tiers as ‘cumulative’, whereby the evidence levels for tier 3 included those in tiers 1 and 2, we’ve put all evidence requirements into a single table for each tier. The evidence requirements have not changed, but this change in presentation is intended to make the ESF easier to use.
3. The functional category that was originally named ‘simple monitoring’ has been renamed ‘health diaries’, to make it easier to distinguish between the functional categories in Tier B: understanding and communicating.
4. We have improved the explanations of which DHTs are likely to fit into which functional category (shown in Table 1 of the ESF document).
5. Links have been added to relevant documents and services, including Healthtech Connect, PHE’s evaluation guide for DHTs, and the NHSX digital health technology standard.

One new supporting document has been published alongside the ESF. This is a checklist diagram to provide a visual guide to using the ESF. This is intended to give a simple outline of how the ESF is intended to be used.

The full ESF and supporting documents are available at https://www.nice.org.uk/about/what-we-do/our-programmes/evidence-standards-framework-for-digital-health-technologies.

### Assessing the impact of the ESF between December 2018 and December 2020

Since its initial publication in December 2018 the NICE ESF has been widely viewed and discussed within the HTA and digital healthcare fields. Here we provide some illustrative examples of the global impact of the ESF.

- The ESF page on the NICE website has been viewed over 55,000 times since publication in December 2018 and downloaded over 19,000 times.
- The most downloaded of the supporting documents are the user guide (over 2500 downloads), the budget impact template (over 1500 downloads) and the functional classification case studies (over 1400 downloads).
- Several healthcare systems, academic groups and commercial organisations outside of the UK have expressed interest in the NICE ESF. These include academic groups, national evaluators or governmental bodies from India, Norway, Indonesia, The Republic of Korea, Denmark, the Netherlands, and Sweden.
- The ESF has been cited in over 50 academic publications.
- The descriptive paper explaining the purpose of the ESF^10^ has been cited 35 times.
- The accelerator groups that were involved in the co-design of the ESF use the standards routinely to advise innovators on evidence generation.
- The ESF is now being used as the standard by innovators seeking to access funding from the UK’s major research and development funders such as NIHR i4i and Innovate UK SME.

## Discussion

We have described the development and update of the NICE ESF, a framework to help guide DHT developers in planning their evidence base, and for commissioners to evaluate the evidence on DHTs. In doing so, we used experience and insights from stakeholders as well as from NICE’s medical technologies evaluation programme (MTEP), from the evaluation of digital mental health technologies for the NICE and NHSE IAPT assessment programme, and incorporated lessons learned from other work on the assessment of DHTs.^5,13–20,23,24^ MTEP was established in 2009 to evaluate medical devices, which similar to DHTs, frequently have low levels of evidence and this experience helped to inform the design of the functional categories. The NICE and NHSE IAPT assessment programme evaluated DHTs that provided therapist-guided psychological therapies. Work from this programme helped to inform the setting of evidence levels, and understanding the context of, and other requirements (such as NHSE clinical safety requirements and local governance at NHS Trusts) for implementing DHTs within the health and care system.

It was clear from our workshop discussions that while stakeholders were well informed and encouraging about using new evaluation approaches for digital tools (such as those more familiar in the field of software development like A-B testing), the majority view was that the evidence standards should be grounded in ‘traditional’ approaches to health technology assessment, using a pragmatic evidence hierarchy that has randomised controlled trials at the top as the best practice standards for the most high risk tools, but allows for less rigorous study designs as minimum standards and in lower tiers. Both in the workshops and during the consultation phase, attention was given to the opportunities DHTs present to harness ‘real-world’ evidence for evaluation. The framework acknowledges that the value of new study designs should continue to be investigated as the potential for using real-world data increases.

Despite the rapid development of the ESF within a short timescale we were able to review an extensive literature base and consult with over 100 stakeholders and their organisations to produce a pragmatic guide for the health and care system. There was limited time within the project to fully test the framework; however, we were encouraged by the supplementary case study work. As part of this, researchers who were not in the core project team were able to apply the functional classification and describe evidence on selected DHTs that was in line with that recommended by our standards. Similarly, there was not time to undertake a full systematic synthesis of all previous work, or to pause development of the framework at key stages and consult widely at each stage. Development required frequent minor modifications prior to a final consultation phase.

The ESF is limited to some extent by the exclusion of DHTs that incorporate adaptive algorithms from the framework, and clearly this is an area for further development work. Here, we define ‘adaptive algorithms’ as those that constantly evolve over time, and so their effectiveness is not fixed at any time. This is in contrast to fixed algorithm DHTs, which are within the remit of the ESF. We define fixed algorithms as those that may have periodic updates but are not constantly changing. We would envision that at the point of commissioning a fixed-algorithm DHT, the commissioner and developer would agree processes for rolling out any such updates.

It is anticipated that as consensus develops on the HTA of DHTs that use adaptive AI, the ESF would be extended to include these. Recent work by the British Standards Institute (BSI) and the Association for the advancement of Medical Instrumentation (AAMI) has set out recommendations for AI in medical devices.^25,26^ NHSX’s AI lab^27^ has been created to address the challenges of safe and ethical adoption of AI-based DHTs within the healthcare system and has a number of workstreams to address these challenges. NHSX, NICE, CQC and MHRA will be jointly working on developing a ‘joined-up’ regulatory and approval system^28^ for AI-based DHTs in the health and care system. This work will be provide a useful means for pulling together the currently fragmented system for regulating and evaluating AI-based DHTs, into a coherent process for the UK health and care system.

Another limitation is that our main focus was on health settings but we recognise the extensive and increasing use of DHTs in social care.

A further (and related) limitation of this work is that it conceptualises DHTs as standalone tools which provide specific functions, rather than as part of services or pathways. Future developments in this area are likely to see DHTs become harder to separate as single entities for evaluation, as they become more integrated within clinical pathways or more embedded within health consumer’s own digital ecosystems as providers such as Apple and Google incorporate health functions across a range of devices and consumer facing services. The ESF was designed to be used for appraising evidence for DHTs being commissioned in the UK health and care system and it is less relevant to technologies that are available directly to public users, such as through app stores.

Since its initial publication in December 2018, the NICE ESF has generated global interest and largely positive feedback from evaluators, DHT developers and academic groups. Positive feedback has been given by app evaluator organisations and governmental groups from several countries have shown an interest in the ESF. This indicates that the evaluation of DHTs has proven challenging to many organisations and that the NICE ESF fills a gap in providing an innovative HTA approach for DHTs. In 2019 NICE ran a pilot project to produce NICE guidance on DHTs, and in 2020 announced that the digital evaluation programme was now open to any DHT in evidence tier 3 of the ESF.^29^ This programme allows a formalised evaluation setting for higher risk DHTs.

In 2019 we sought and responded to feedback on the ESF and made minor amendments to the ESF in response. We acknowledge the need for continual surveillance of the digital healthcare field over time and anticipate further iterations of the ESF in coming years.

## Conclusions

The NICE evidence standards framework for digital health and care technologies demonstrates how a novel approach to HTA can be taken in a fast-moving field, whereby proportionality provides a pragmatic solution to the need for an agile approach. It requires higher levels of evidence for the functions that pose the highest risk to health, and more intensive health economic analysis when the financial risk to the health system is highest. This framework is designed to support local and national purchasing decisions around DHTs in England, and to help developers of DHTs to plan the generation of their evidence base.

The ESF was developed in a dynamic way. It was informed by the existing literature but also through broad and iterative consultation with healthcare commissioners and healthcare innovators, creating a co-design approach. We have continued to closely monitor the digital healthcare environment in England and have used stakeholder feedback to ensure that the ESF is up to date and meets the needs of users. We believe that the ESF may also provide a useful template for other jurisdictions and health systems that are considering ways to evaluate DHTs.
